# Effect of Transcriptional Regulatory Factor FoxO3a on Central Nervous System Oxygen Toxicity

**DOI:** 10.3389/fphys.2020.596326

**Published:** 2020-12-18

**Authors:** Yanan Zhang, Benming You, Yuliang Chen, Junlin Yang, Chengwei Xie, Guoyang Huang, Runping Li, Ping Hu

**Affiliations:** ^1^Department of Diving and Hyperbaric Medicine, Naval Special Medical Center, Navy Medical University, Shanghai, China; ^2^Department of Pharmacy, Changhai Hospital, Navy Medical University, Shanghai, China; ^3^Department of Nautical and Aviation Medicine Center, The Sixth Medical Center of Chinese PLA General Hospital, Beijing, China; ^4^State Key Laboratory of Cell Biology, Center of Excellence in Molecular and Cell Biology, Shanghai Institute of Biochemistry and Cell Biology, Chinese Academy of Sciences, Shanghai, China; ^5^Institute for Stem Cell and Regeneration, Chinese Academy of Sciences, Beijing, China; ^6^Bioland Laboratory (Guangzhou Regenerative Medicine and Health Guangdong Laboratory), Guangzhou, China; ^7^Xinhua Hospital, Shangahai Jiao Tong University, Shanghai, China; ^8^Shanghai Institute of Stem Cell Research and Clinical Translation, Shanghai, China

**Keywords:** hyperbaric oxygen, oxygen toxicity, central nervous system, FoxO3a, oxidative stress

## Abstract

Central nervous system (CNS) oxygen toxicity (CNS-OT) is a toxic reaction that appears after the inhalation of gas at an excessive oxygen partial pressure during underwater operation or hyperbaric oxygen (HBO) treatment. The mechanism of CNS-OT has not been clearly characterized. Though it has been attributed to the excessive oxidative stress induced by HBO, evidences against this hypothesis have been reported. Here we find that Forkhead box protein O3 (FoxO3a) is important for CNS-OT protection. FoxO3a knock-out (KO) mice had a shorter latency to develop convulsions and greater number of seizures within a certain period of time. The acute lung injury (ALI) induced by CNS-OT was also more severe in FoxO3a KO mice. Further analysis reveals a significant decrease in the activity of catalase (CAT), an antioxidant enzyme and a significant increase in the content of malondialdehyde (MDA), an oxidative product, in brain tissues of FoxO3a KO mice. Short-time HBO exposure could increase FoxO3a expression level and trigger its nuclear translocation. The level of nuclear localized FoxO3a peaked at 8 h after exposure. Our results demonstrate that the activity of FoxO3a is highly sensitive to HBO exposure and FoxO3a plays important roles in protecting CNS-OT. Further mechanic analysis reveals that FoxO3a protects CNS-OT via activating antioxidative signaling pathway.

## Introduction

Hyperbaric oxygen (HBO) is widely applied in underwater missions, hyperbaric facility operation and disease treatment. However, after inhalation of the gas with high oxygen partial pressure (OPP) for time beyond a limit, a toxic reaction, i.e., oxygen toxicity (OT), occurs. When the OPP is greater than 3 ATA, OT is characterized by central nervous system (CNS) dysfunction as the main manifestation, called CNS-OT, and the most typical and intense manifestation is grand mal seizure, that is, “oxygen convulsion” (Zhu et al., 2016; Wingelaar et al., 2017; Ciarlone et al., 2019). At present, the exact pathogenesis of oxygen convulsion remains to be explored. The currently available preventive measures are to restrict OPP and shorten the oxygen inhalation duration. The protective effects of these measures are limited.

A large number of studies have shown that various oxygen free radical-producing links in the body are activated during HBO exposure, which can generate massive reactive oxygen species (ROS) and reactive nitrogen species (RNS), including O_2_^–^, H_2_O_2_, NO, ONOO^–^, and OH. The overproduced ROS and RNS can inhibit the activities of various antioxidant enzymes, consume large amount of reducing reagents and increase the content of various oxidative products, therefore disrupting the redox balance *in vivo*, and leading to structural and functional damage of cells and tissues (Chavko et al., 2003; Poff et al., 2017; Ciarlone et al., 2019). Inhibition of ROS and RNS production and bringing back the redox balance *in vivo* are considered to be important strategies to prevent OT. However, supplement of anti-oxidative reagents does not show the protective effects as expected (Boadi et al., 1991; Dennog et al., 1999; Bader et al., 2007), suggesting that the functions of ROS and RNS in CNS-OT needs to be further studied and key molecules for effective CNS protection needs to be identified. Identification of the key molecule for CNS-OT protection will help us understanding the mechanism better and develop new strategy to prevent CNS-OT.

Forkhead box protein O3, also known as FoxO3 or FoxO3a, is a human protein encoded by the FoxO3 gene and is widely distributed in multiple organs, including muscles, CNS, peripheral nerves, stomach, eyes, heart, and lungs. In the CNS, it is widespread in the cerebral cortex, hippocampus, as well as cerebellum. Studies have shown that it can play an important role in inhibiting tumor cell proliferation, cell cycle progression, promoting apoptosis, oxidative stress and prolonging lifespan through phosphorylation, polyubiquitinated degradation, acetylation or deacetylation, as well as microRNA modalities (Ni et al., 2014; Liang et al., 2017; Codogno and Morel, 2018; Fasano et al., 2019; Zhou et al., 2019; Usami et al., 2020). Some studies have indicated that it is a transcriptional regulator closely related to oxidative stress in cells and plays a very important role in oxidative stress. A variety of oxidative stimuli can induce FOXO3a transcriptional activation and promote the up-regulation of antioxidant enzyme genes (e.g., MnSOD, Catalse, etc.), which in turn scavenges ROS and reduces peroxidation damage. However, in different stimulation types and under different pathophysiological states, the intensity of its regulatory effect and the specific mechanism need to be further investigated deeply (Sundaresan et al., 2009; Rangarajan et al., 2015; Zhou et al., 2019).

The function of FoxO3a in CNS-OT seizures has not been characterized. Here we used FoxO3a gene knock-out (KO) mice to investigate the function of FoxO3a in pathogenesis and oxidative damage of CNS-OT. We found that FoxO3a translocated to nuclei and the expression level of FoxO3 also increased in cerebral cortex and lung tissue upon HBO inhalation. Knocking out FoxO3a led to a shorter latency to develop convulsions, greater number of seizures, and more severe acute lung injury (ALI) induced by CNS-OT. A significant decrease in the activity of catalase (CAT), an antioxidant enzyme and a significant increase in the content of malondialdehyde (MDA), an oxidative product, in brain tissues of FoxO3a KO mice, suggested that FoxO3a functions through its anti-ROS activity. In summary, we identified FoxO3a as an important protective factor for CNS-OT upon HBO inhalation.

## Materials and Methods

### Animals

All procedures were performed in accordance with Navy Medical University (NMU) Guide for the care and use of laboratory animals, and approved by the ethics committee for Animal Experiments of NMU.

Adult male FoxO3a gene knock-out FVB mice (FoxO3a KO mice) and wild type FVB mice (WT mice) were provided by Institute of Biochemistry and Cell Biology, Chinese Academy of Sciences (Shanghai, China). Adult male C57BL/6 mice weighing 22∼26 g, were purchased from Xipuer-Bikai laboratory animal Co. Ltd. (Shanghai, China).

The breeding environment was maintained at a temperature of 22 ± 1°C and a humidity of 40–50%, and daylight hours were set to 12 per day. Animals were allowed free access to food and drinking water.

### Part 1: Study on the Incidence of CNS-OT in FoxO3a KO Mice

#### Animal Groups

Wild type mice were randomly divided into two groups: normal group, no treatment with HBO; HBO-exposed group 1, being exposed to HBO at 6 ATA for 30 min. Besides, HBO-exposed group 2 was set with FoxO3a KO mice, similarly exposed to HBO at 6 ATA for 30 min. There were 12 mice in each group.

#### Measurement of Oxygen Convulsion Latency and Number of Seizures Within 30 min

Mice in HBO-exposed groups 1 and 2 were placed in the experimental HBO chamber (RDC150-300-6, NMU, Shanghai, China). Firstly the chamber was flushed with pure oxygen at a flow rate of 1 L/min for 5 min to replace the air inside. The oxygen concentration in the chamber was monitored with an oximeter, and after the oxygen concentration was >99%, the chamber pressure was increased to 6 ATA with pure oxygen at a rate of 1 ATA/min. During pressurization and high pressure dwell, ventilation was continued at a flow rate of 0.3 L/min and the CO_2_ concentration inside the chamber was controlled by placing soda lime in it. At the end of the exposure, the pressure was reduced to normal atmosphere at the rate of 1 ATA/min. The period from the time when the chamber pressure reached 6 ATA to the first onset of grand mal seizures such as generalized tonic-clonic seizure was calculated as the oxygen convulsive latency. The whole HBO exposure lasted for 30 min, and the number of convulsions occurring within 30 min was recorded.

#### Observation of Changes in Lung Tissue Structure

At the end of HBO exposure, mice were anesthetized with pentobarbital and sacrificed by cervical dislocation. Lung tissues were taken, rinsed, fixed, and stained, and the changes in lung tissue structure were observed under a light microscope. Four mice were set per group.

#### Apoptosis Detection by Tunel Staining

At the end of HBO exposure, the mice were anesthetized with pentobarbital and cannulated through the left atrial appendage. The blood cells in the pulmonary vessels were flushed with saline, and the tissues were fixed by perfusion with 4% paraformaldehyde. Lung tissues were taken and Tunel staining was performed according to the assay kit instructions (Roche, Switzerland). Under a light microscope, four rectangular regions (400×) were randomly selected and 100 cells were counted in each region to calculate the percentage of Tunel-positive cells within each region. Four mice were set per group.

#### Determination of Water Content in Lung Tissues

At the end of HBO exposure, mice were anesthetized with pentobarbital and sacrificed by cervical dislocation. One side of lung tissues were harvested, the water on the surface was dried, followed by weighing and then drying at 60°C for more than 72 h. After stabilizing to a constant weight, the water content in lung tissues was calculated. Water content (%) = (wet weight - dry weight)/wet weight × 100%. Eight mice were set per group.

#### Determination of Total Protein Content and LDH Activity in Alveolar Lavage Fluid

At the end of HBO exposure, the mice were anesthetized with pentobarbital. The alveoli were lavaged with 1.0 ml of pre-cooled saline, and alveolar lavage fluid was collected to detect the total protein content (μg/μl) and LDH activity (U/g prot) in them. Eight mice were set per group.

#### Determination of H_2_O_2_ and MDA Contents, CAT and MnSOD Activities in Cerebral Cortex and Lung Tissues

At the end of HBO exposure, the mice were anesthetized with pentobarbital and sacrificed by cervical dislocation, and the cerebral cortex and lung tissues were harvested. The tissues were lysed with lysis buffer, centrifuged, and the supernatant was taken for testing. The contents of H_2_O_2_ and MDA, the activities of CAT and MnSOD, as well as the protein content were measured, respectively, according to the instructions of the assay kit (Beyotime, China). The results were expressed as the corresponding amount of the protein per unit weight (μmol/g prot or U/mg prot). Eight mice were set per group.

### Part 2: Effect of HBO on FoxO3a Protein Expression in Brain and Lung Tissues

#### Animal Grouping and HBO Exposure

The C57BL/6 mice were randomly divided into four groups: Normal group, no exposure treatment; HBO-exposed Group 1, samples were taken immediately after the end of exposure; HBO-exposed group 2, samples were taken 8 h after the end of exposure; HBO-exposed group 3, samples were taken 24 h after the end of exposure. All exposed mice were exposed to HBO at 6 ATA for 30 min in the same manner as described above.

#### Determination of FoxO3a Protein Content in Tissues

The mice were anesthetized with pentobarbital and sacrificed by cervical dislocation, and the cerebral cortex and lung tissues were taken. The tissues were lysed with lysis buffer and centrifuged. The supernatant was taken to detect the FoxO3a protein content by Western blotting. The primary antibody was rabbit monoclonal to FoxO3a (Abcam), and the secondary antibody was HRP-labeled rabbit monoclonal to beta Actin (Abcam, United States). The ECL luminescence reagent was used for coloring, the fluorescence imaging instrument was used to record and analyze images, and the Image J 2X software was used to analyze gray values. Three mice were set per group.

#### Determination of FoxO3a mRNA Content in Tissues

The mice were anesthetized with pentobarbital and sacrificed by cervical dislocation, and cerebral cortex and lung tissues were taken to detecting the FoxO3a mRNA content by RT-PCR. The total RNA was extracted following the kit instructions (Trizol Reagent, Invitrogen) and subjected to reverse transcription as indicated (RevertAid First Strand cDNA Synthesis Kit, Thermo). PCR amplification was performed using the FastStart Universal SYBR Green Master kit (Rocher). MxPro-Mx3005P 4.1 software was used to analyze the data. Four mice were set per group. Primer sequences were:

FoxO3a, Forward: 5′-GGCAACCAGACACTCCAAGAC-3′

Reverse, 5′-GGTGGTGGAGCAAGTTCTGATT-3′

β-actin, Forward: 5′-GTGACGTTGACATCCGTAAAGA-3′

Reverse: 5′-GTAACAGTCCGCCTAGAAGCAC-3′

#### Examination the Distribution of FoxO3a in the Cytoplasm and Nucleus

At the end of HBO exposure, the mice were anesthetized with pentobarbital and cannulated through the left atrial appendage. The blood cells in the pulmonary vessels were flushed with saline, and the tissues were fixed by perfusion with 4% paraformaldehyde. Lung tissues were obtained and the protein content of FoxO3a in the tissues was measured by immunohistochemistry. The primary antibody was rabbit monoclonal to FoxO3a (Abcam), and the secondary antibody was HRP-labeled rabbit monoclonal to beta Actin (Abcam, United States). DAB was used for coloring, and image data were analyzed by Image pro-plus 6.0 software. Four mice were set per group.

#### Statistical Methods

Statistical analysis was performed using SPSS 17.0 software, and the results obtained were expressed as mean ± standard deviation (X ± SD). Independent sample *T* test was used for sample comparison between two groups; one-way ANOVA was used for homogeneity test of variance for sample comparison across multiple groups, and intergroup differences were analyzed, followed by LSD test and Dunnett *T* test for pairwise comparison. *P* < 0.01 indicated very significant difference, *P* < 0.05 indicated significant difference, and *P* > 0.05 indicates no significant difference.

## Results

### Effect of FoxO3a Deletion on Convulsion Latency and Number of Seizures

**FIGURE 1:**
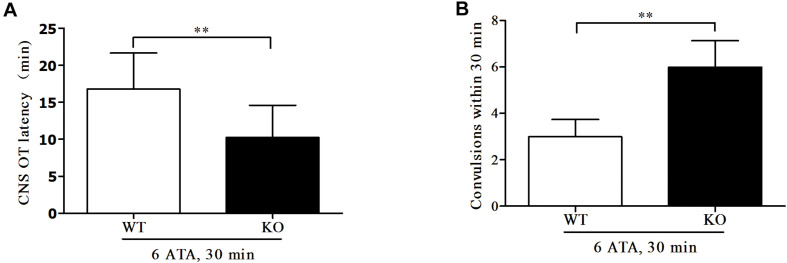
Effect of FoxO3a deletion on oxygen convulsive latency and number of convulsive seizures. Mice in HBO-exposed groups 1 and 2 were placed in the experimental HBO chamber. The first onset of grand mal seizures such as generalized tonic-clonic seizure was calculated as the oxygen convulsive latency **(A)**; The number of convulsions occurring within 30 min was recorded **(B)**. **P < 0.01. *n* = 12.

Compared with WT mice, the convulsive latency of FoxO3a KO mice was significantly shortened and the number of convulsive seizures within 30 min of exposure was significantly increased (Figure 1).

### Effect of FoxO3a Deletion on Acute Lung Injury Induced by Oxygen Convulsion

**FIGURE 2:**
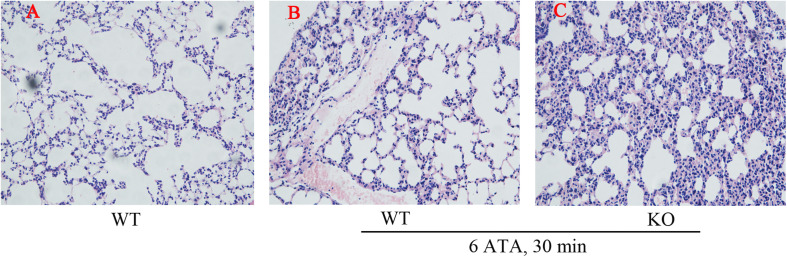
Effect of FoxO3a deletion on histopathology associated with acute lung injury induced by oxygen convulsion. The lung tissue structure were observe by the Hematoxylin-Eosin (HE) staining. Original magnification: ×200. *n* = 4. **(A)** The group of HBO-unexposed WT mice, **(B)** the group of HBO-exposed WT mice, and **(C)** the group of HBO-exposed FoxO3a KO mice.

Histopathological examination of the lungs showed that, compared with WT mice without HBO exposure, both HBO-exposed WT mice and FoxO3a KO mice developed lung injury characterized by hemorrhage, and FoxO3a KO mice had more severe congestion in the bronchiolar wall and alveolar tissues, more inflammatory cell infiltration, and more disorganized lung tissue structures (Figure 2).

**FIGURE 3:**
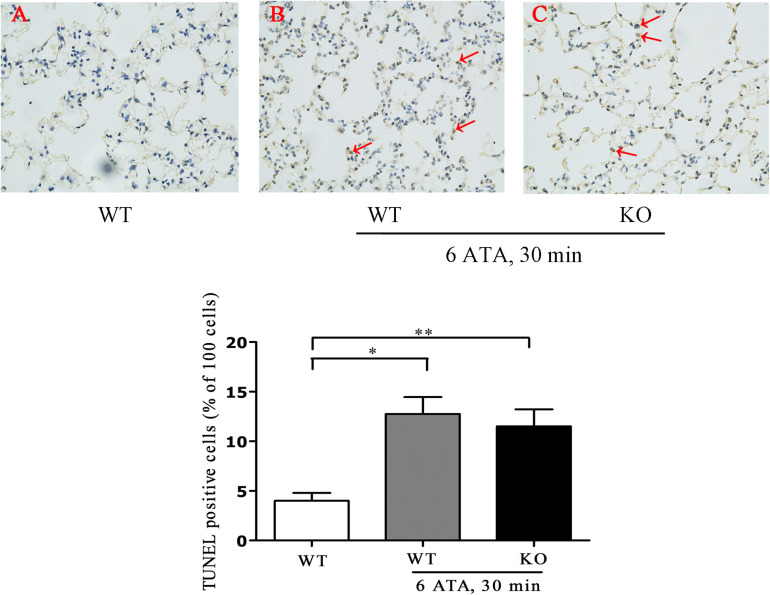
Effect of FoxO3a deletion on apoptosis associated with acute lung injury induced by oxygen convulsion. Lung tissues were taken and Tunel staining was performed according to the assay kit instructions. **P < 0.01, *P < 0.05. *n* = 4. **(A)** The group of HBO-unexposed WT mice, **(B)** the group of HBO-exposed WT mice, and **(C)** the group of HBO-exposed FoxO3a KO mice. Red arrow indicate the apoptotic cells.

Tunel assay results showed that the positive rate of apoptotic cells significantly increased in both HBO-exposed WT mice and FoxO3a KO mice compared with WT mice without HBO exposure, but there was no statistically significant difference in the positive rate between the two exposure groups (Figure 3).

**FIGURE 4:**
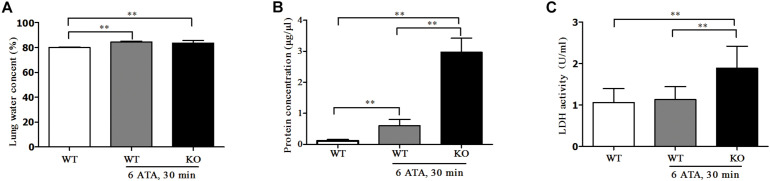
Effect of FoxO3a deletion on tissue edema of lung, protein and LDH exudation associated with acute lung injury induced by oxygen convulsion. Water content (%) = (wet weight–dry weight)/wet weight × 100% **(A)**; Protein concentration **(B)** and LDH activity **(C)** in BALF were tested by enzyme-linked immunosorbent assay (ELISA). **P < 0.01. *n* = 8.

Compared with HBO-unexposed WT mice, both HBO-exposed WT mice and FoxO3a KO mice showed significantly increased water content in lung tissues. This was a sign of edema, but there was no significant difference in water content between the two exposure groups (Figure 4A). The content of total protein in the alveolar lavage fluid also significantly increased, and the increase was more pronounced in KO mice than in WT mice (Figure 4B). The LDH activity in the alveolar lavage fluid of HBO-exposed WT mice did not change significantly, but LDH activity significantly increased in exposed FoxO3a KO mice (Figure 4C).

The above results indicated that after FoxO3a gene deletion, oxygen convulsions could lead to more severe ALI.

### Effect of FoxO3a Deletion on the Content of Oxidative Products in the HBO-Exposed Cortex and Lung Tissues

**FIGURE 5:**
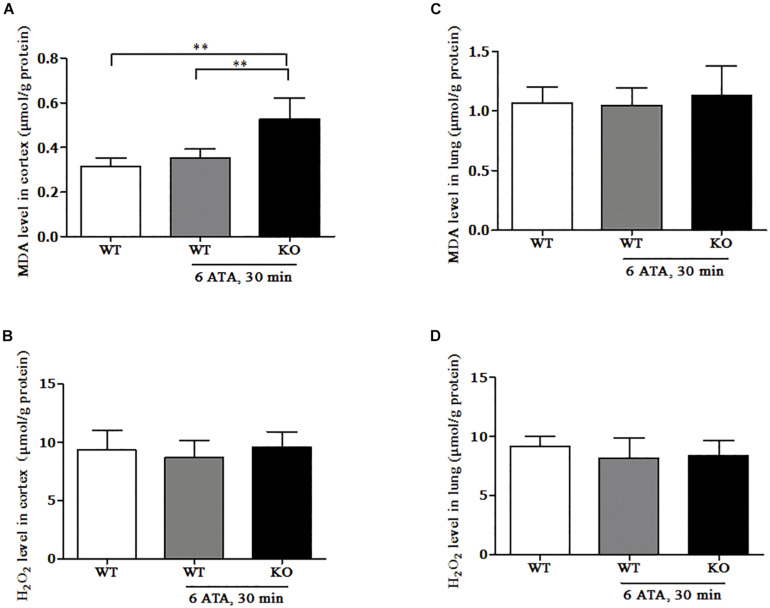
Effect of FoxO3a deletion on the content of oxidative products in the HBO-exposed brain and lung tissues. Determination of H_2_O_2_ and MDA contents in cerebral cortex **(A,B)** and lung tissues **(C,D)** were tested by enzyme-linked immunosorbent assay (ELISA). **P < 0.01. *n* = 8.

In the cerebral cortex, compared with that of WT mice without HBO exposure, the MDA content of WT mice exposed to HBO showed no significant change, but significantly increased in FoxO3a KO mice after HBO exposure (Figure 5A). There was no significant difference in H_2_O_2_ content between the exposed groups and the non-exposed group (Figure 5B). In the lung tissues, the MDA and H_2_O_2_ contents showed no significant differences between the exposed groups and the non-exposed group (Figures 5C,D).

### Effects of FoxO3a Deletion on Antioxidant Enzyme Activities in HBO-Exposed Cortex and Lung Tissues

**FIGURE 6:**
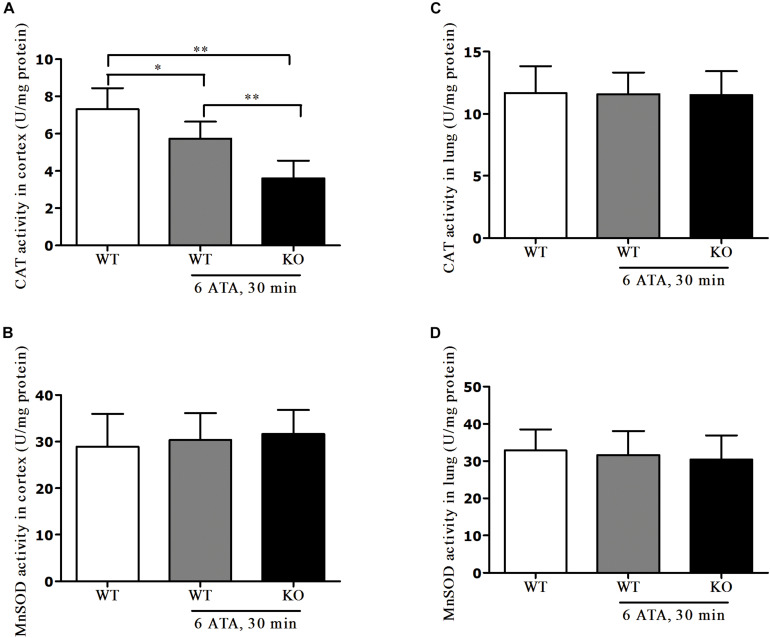
Effects of FoxO3a deletion on antioxidant enzyme activities in HBO-exposed brain and lung tissues. Determination of CAT activity and MnSOD activity in cerebral cortex **(A,B)** and lung tissues **(C,D)** were tested by enzyme-linked immunosorbent assay (ELISA). **P < 0.01, *P < 0.05. *n* = 8.

In the cerebral cortex, compared with that of WT mice without HBO exposure, CAT activity significantly decreased in both WT and FoxO3a KO mice after exposure but the decrease was more pronounced in FoxO3a KO mice (Figure 6A). There was no significant difference in MnSOD activity between the exposed groups and the non-exposed group (Figure 6B). In lung tissue, there were no significant differences in CAT and MnSOD activities between the exposed groups and the non-exposed group (Figures 6C,D).

### Effect of HBO Exposure on FoxO3a Protein Expression in Cerebral Cortex

**FIGURE 7:**
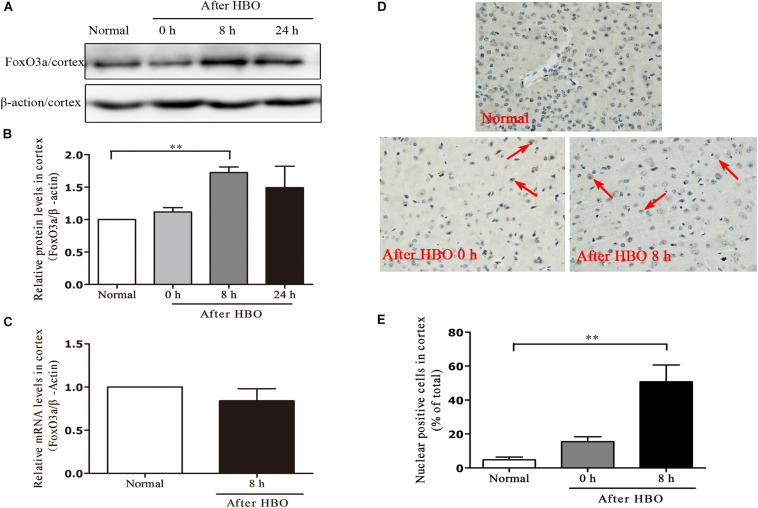
Effect of HBO exposure on FoxO3a protein expression in cerebral cortex. Western blot results of FoxO3a protein expression at different time points after exposure **(A)**; Content of FoxO3a protein at different time points after exposure **(B)**; mRNA content of FoxO3a 8 h after the end of exposure **(C)**; Cells positive for FoxO3a expression at different time points after exposure as detected by immunohistochemistry **(D)**; Results of counting cells with positive expression of FoxO3a in the nuclei at different time points after exposure **(E)**. **P < 0.01. *n* = 4. Red arrow indicate the FoxO3a nuclear entry cells.

In the cerebral cortex, as short as 30 min of 6 ATA HBO exposure could initiate enhanced FoxO3a protein expression, and the level peaked at 8 h after the end of exposure (Figures 7A,B). Compared with the normal group without HBO exposure, the FoxO3a mRNA content at 8 h after exposure did not show a significant change (Figure 7C). HBO exposure also induced a significant transfer of FoxO3a from the cytoplasm to the nucleus, and again at 8 h after exposure, the phenomenon of nuclear entry was most pronounced (Figures 7D,E).

### Effect of HBO Exposure on FoxO3a Protein Expression in Lung Tissues

**FIGURE 8:**
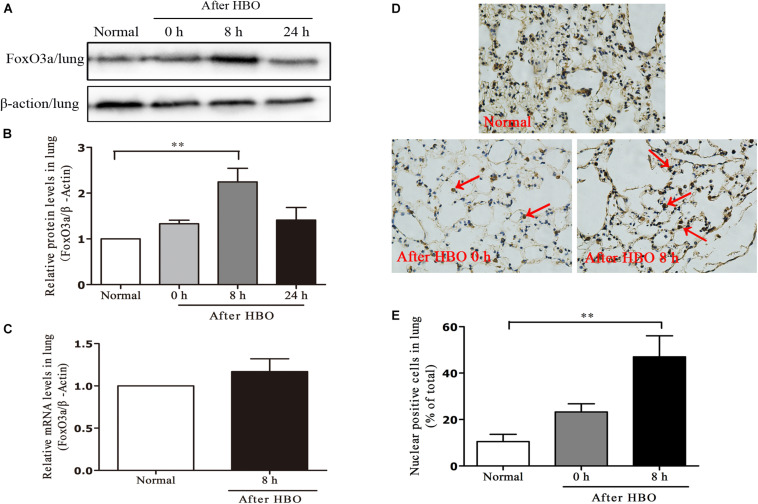
Effect of HBO exposure on FoxO3a protein expression in lung tissues. Western blot results of FoxO3a protein expression at different time points after exposure **(A)**; Content of FoxO3a protein at different time points after exposure **(B)**; mRNA content of FoxO3a 8 h after the end of exposure **(C)**; Cells positive for FoxO3a expression at different time points after exposure as detected by immunohistochemistry **(D)**; Results of counting cells with positive expression of FoxO3a in the nuclei at different time points after exposure **(E)**. **P < 0.01. *n* = 4. Red arrow indicate the FoxO3a nuclear entry cells.

In lung tissues, the results were similar to those in cerebral cortex. The FoxO3a protein content peaked at 8 h after HBO exposure (Figures 8A,B), but HBO exposure did not cause a significant change in the mRNA content of FoxO3a (Figure 8C). The amount of FoxO3a nuclear entry also peaked at 8 h after the end of exposure (Figures 8D,E).

## Discussion

At present, our understanding on the pathogenesis of CNS-OT and its prevention measures has been very limited. It is mainly considered that HBO exposure can lead to the increase of oxygen content in tissues, far exceeding the capacity of mitochondria to completely reduce it to H_2_O molecules through cellular respiration. Due to the presence of excessive amount of oxygen, the respiration is over activated. The overactivation of respiration produces massive amount of ROS, including O_2_^–^, OH, H_2_O_2_, etc. In addition, increased oxygen content in tissues can also activate the NO-generating pathway, which ultimately leads to a significant increase in the content of RNS, such as NO and OONO^–^. A large increase in ROS and RNS can induce a series of strong oxidative stress reactions, leading to oxidative damage, production of various oxidative products such as MDA, and ultimately OT symptoms. At the same time, ROS and RNS can also inhibit the activity of antioxidant enzymes in the body, including SOD and CAT as well as a large number of substances with antioxidant activity in the body, such as GSH and Prdx. This would further destroy the antioxidant system in the body, and ultimately aggravate the oxidative damage of cells, tissues and organs (Poff et al., 2017; Yu et al., 2017; Ciarlone et al., 2019). Improving the antioxidant capacity of the body, supplementing more anti-oxidative substances, and reducing the production of oxygen free radicals, and their subsequent effects are considered to be important strategies for the prevention of CNS-OT. However, it has been reported that supplement of anti-oxidative substances failed to show protective effects on CNS-OT, suggesting more complicated mechanism is involved (Boadi et al., 1991). Here we showed that FoxO3a plays an important role in preventing CNS-OT.

Forkhead box protein O3 has been shown to play important roles in regulating cell proliferation, differentiation, metabolism, apoptosis, DNA damage repair, life span, and oxidative stress responses (Fasano et al., 2019). It is one of the key transcription factors regulating oxidative stress signaling pathway. For example, the antioxidant DMY can protect HUVECs from SNP-induced oxidative damage by activating the PI3K/Akt/FoxO3a signaling pathway (Zhang et al., 2019); PPQ can protect HK-2 from high glucose-induced oxidative damage and apoptosis through Sirt3 and PI3K/Akt/FoxO3a signaling pathways (Wang et al., 2018); and TSA protects myocardium from oxidative stress-mediated damage by increasing H4 acetylation in the FoxO3a promoter region as well as the expression of FoxO3a, MnSOD, and CAT (Guo et al., 2017). However, it has also been reported that FoxO3a can instead induce apoptosis under oxidative stress conditions in the body. For example, during oxidative stress and apoptosis induced by high glucose in CMECs, the activation of FoxO3a promotes the occurrence of apoptosis (Peng et al., 2013); during oxidative stress induced by H_2_O_2_ in PC12, the phosphorylation level of FoxO3a is significantly reduced, resulting in significant apoptosis of the cells (Safarian et al., 2015). It can be seen that FoxO3a can not only protect the survival of cells under oxidative stress conditions, but also induce apoptosis, which may be related to its diverse functions through different mechanisms in different tissues, cells, and under different oxidative stress conditions.

We used FoxO3a KO mice to investigate the status of grand mal seizures and ALI triggered by CNS-OT for the purpose of assessing the role of FoxO3a in the development of CNS-OT. The results of the study showed that after FoxO3a deletion *in vivo*, the development of CNS-OT seizures was faster, as shown by a shorter convulsive latency. The severity of the disease was higher, as shown by an increase in the number of seizures over a certain period of time. Besides, the onset of CNS-OT can cause ALI through routes such as activating sympathetic nerves and leading to pulmonary vascular hypertension. ALI is mainly manifested as pulmonary hemorrhage and protein exudation etc., which can cause dyspnea, hypoxia, and even death within a short period of time (Demchenko et al., 2008, 2011, 2012). As a very important concomitant effect in the onset of CNS-OT, ALI has become an important reference index for the investigation of the severity of CNS-OT onset and the study of its pathogenesis and effectiveness of prophylaxis and treatment measures. In our study, histopathological examination of lung showed that without FoxO3a, mice had more severe congestion in the bronchiolar wall and alveolar tissues, more inflammatory cell infiltration, and more disorganized lung tissue structures. The content of total protein in the alveolar lavage fluid was more pronounced, and the LDH activity in it also significantly increased. All these results indicated an aggravation of the degree of ALI. Based on our all above findings, we can conclude that FoxO3a has a very important role in counteracting CNS-OT episodes.

Further examination of oxidative stress-related parameters showed that after FoxO3a deletion, HBO exposure resulted in lower activity of antioxidant enzyme CAT and higher content of oxidation product MDA in brain tissues, indicating that FoxO3a could indeed participate in the occurrence of CNS-OT through its regulated oxidative stress pathway, and also further confirmed the important role of redox balance system *in vivo* in counteracting the onset of CNS-OT.

As a transcriptional regulator, when FoxO3a accumulates in the nucleus, it can exert its transcriptional activity. At this time, FoxO3a can bind to the promoters of some important anti-oxidative stress genes (such as MnSOD gene and Catalse gene) and activate their expression, which plays an important role to down-regulate ROS levels and protect the body against oxidative stress damage. For example, during H_2_O_2_-induced oxidative stress in hepatocytes, vein-derived Eckol can phosphorylate FoxO3a by inducing AMPK, prompting its entry into the nucleus, ultimately increasing the expression of intracellular MnSOD and protecting cells from oxidative stress damage (Kim et al., 2014). In H_2_O_2_-induced cell damage, NAD^+^-dependent SIRT2 can enter the nucleus to bind to FoxO3a and deacetylate it, which in turn increases its binding to DNA, elevates the expression of its target gene MnSOD, etc., and ultimately promotes the survival of cells (Wang et al., 2007). So, the process of FoxO3a entering the nucleus is a key link in its antioxidant transcriptional regulation.

Our study found that when the OPP was high enough (6 ATA), the nuclear entry and protein expression process of FoxO3a could be initiated with a short time of exposure (30 min). This showed that FoxO3a had a high sensitivity to this oxidative stimulus by HBO exposure, and it would indeed actively participate in counteracting the oxidative damage caused by HBO to the body. This is a further demonstration that it has a close relationship with OT, and its absence would induce and aggravate the toxic effects of HBO.

Enhancing the activity of antioxidant enzymes *in vivo*, increasing the content of antioxidant substances, and keeping the body sufficiently high antioxidant capacity are important strategies for the prevention of OT (Poff et al., 2017; Ciarlone et al., 2019). The antioxidant defense system *in vivo* is a complex tissue, and all links are very important when the body is subject to strong oxidative stimuli, from upstream signal regulation to downstream antioxidant enzymes and substances playing their roles (Vilhardt et al., 2017; Snezhkina et al., 2019). For different inducements and types of oxidative stimuli, and the different results and diseases caused by them, in-depth analysis on the role of each link in the oxidative stress pathway is particularly important for revealing the pathogenesis of the disease and finding the effective countermeasures. Our study demonstrates for the first time that FoxO3a has high responsiveness to the oxidative stimulus of HBO exposure; it has a very important impact on the onset of CNS-OT, and is involved its regulation of targets and pathways such as downstream antioxidant enzymes. Focusing on the regulatory role of FoxO3a in oxidative stress signal transduction pathways, as well as factors and substances affected by its regulatory role, further in-depth investigation of the mechanism of FoxO3a in the development of CNS-OT, as well as its function in various effects of the body induced by HBO will be helpful to more comprehensively and accurately reveal the causes of CNS-OT and expand the scope of finding effective countermeasures; this will also help us to more comprehensively understand the various effects of HBO on the body including its therapeutic effects.

## Data Availability Statement

The raw data supporting the conclusions of this article will be made available by the authors, without undue reservation.

## Ethics Statement

The animal study was reviewed and approved by the Ethics Committee for Animal Experiments of Navy Medical University.

## Author Contributions

RL, PH, YZ, and BY designed the experiments, wrote the manuscript, and prepared all the figures. YZ, BY, YC, and CX conducted the experiments. YZ, BY, YC, JY, CX, GH, RL, and PH contributed to data analyses and interpretation of the results. YC, JY, CX, and GH revised the manuscript. All authors contributed to the article and approved the submitted version.

## Conflict of Interest

The authors declare that the research was conducted in the absence of any commercial or financial relationships that could be construed as a potential conflict of interest.
